# Editorial: Ying and Yang Members of the Tumor Necrosis Factor Superfamily: Friends or Foes in Immune-Mediated Diseases and Cancer

**DOI:** 10.3389/fimmu.2017.01584

**Published:** 2017-11-23

**Authors:** Adebola Giwa, Rizwan Ahmed, Thomas Donner, Hideo Yagita, Abdel Rahim A. Hamad

**Affiliations:** ^1^Department of Pathology, Johns Hopkins University School of Medicine, Baltimore, MD, United States; ^2^Department of Pediatrics, Johns Hopkins University School of Medicine, Baltimore, MD, United States; ^3^Department of Medicine, Johns Hopkins University School of Medicine, Baltimore, MD, United States; ^4^Department of Immunology, Juntendo University School of Medicine, Tokyo, Japan

**Keywords:** Fas pathway, type 1 diabetes, CD95L, FasL, tumor necrosis factor

**Editorial on the Research Topic**

**Ying and Yang Members of the Tumor Necrosis Factor Superfamily: Friends or Foes in Immune-Mediated Diseases and Cancer**

Tumor necrosis factor (TNF) and TNF-receptors (TNFR) superfamilies represent one of the earliest immunological systems to be targeted for immunotherapy as typified by the use of anti-TNFα agents for treating Crohn’s disease in patients refractory to corticosteroids and conventional immunosuppressives (1). It is also one of the largest. There are more than 18 receptor/ligand pairs in the TNF/TNFR superfamily and many, if not most of them, are currently being evaluated as immunotherapeutic targets in the fields of autoimmunity and cancer. While promising, these efforts are challenged by the complex and intertwining roles various members play in regulating immune homeostasis, proliferation, differentiation, and apoptosis of various cell types. These promises and hurdles are discussed in the following articles published on this research topic, which are focused mainly on Fas/FasL and briefly summarized below (Balomenos et al.; Chakrabandhu and Hueber; Roberts et al.; Saxena et al.; Volpe et al.; Yamada et al.; Yolcu et al.; O’Reilly et al.). Unfortunately, other critical members of the TNF superfamily, including CD40L/CD40, GITR, BAFF, APRIL, TACI, and GITR were beyond the scope of this research topic. However, we refer the reader to scholarly reviews about other members of TNF/TNFR superfamilies that have not been covered here, including their roles in rheumatic diseases (2), neuroinflammation/autoimmunity (Sonar and Lal), and general aspects of their signaling pathways (3).

The articles published under this research topic help to provide a better understanding of the roles of some of the major members of the TNF/TNFR superfamily in the pathogenesis of several diseases and generate considerable hope that their manipulation could lead to therapeutic interventions:

Hamad’s paper on the “Expansion of FasL-Expressing CD5+ B Cells in Type 1 Diabetes Patients” discusses a newly identified subpopulation of FasL+ CD5+ CD19+ B cells that was significantly elevated in Type 1 Diabetes (T1D) subjects. In contrast, to IL-10+ CD5+ B cells that do not express FasL but produce IL-10, FasL+ CD5+ cells do not produce cytokines and are more resistant to activation-induced cell death (AICD). These intriguing findings could lead to better understanding of the mechanisms underlying the pathogenic role of FasL in T1D and cell types expressing it (4, 5).

Yamada’s group’s paper on the “Dual Role of Fas/FasL-Mediated Signal in Peripheral Immune Tolerance” uses data from research studies on Fas/FasL gene mutations in mice and humans to discuss the dual function of the Fas pathway signaling leading to apoptosis or cell proliferation of immune cells depending on local environmental circumstances, and how mutations and disruptions of this process leads to the pathogenesis of autoimmunity (Yamada et al.; Hamad et al.).

Yolcu’s paper on “Fas/Fas-Ligand Interaction As a Mechanism of Immune Homeostasis and β-Cell Cytotoxicity: Enforcement Rather Than Neutralization for Treatment of Type 1 Diabetes” focuses on the immunogenic activities of Fas/Fas-L interactions, as well as how TNF functions in both apoptosis and growth signaling, thereby regulating both immune reactions and injury repair. The paper further details how these processes may be involved in the pathogenesis of T1D and presents arguments for and against therapeutic neutralization of Fas and/or FasL to potentially abrogate T1D (Yolcu et al.).

Balomenos’s paper, “On How Fas Apoptosis-Independent Pathways Drive T Cell Hyperproliferation and Lymphadenopathy in *lpr* Mice,” discusses the role of Fas/FasL and p21 interactions and their effect on T cell apoptosis, hyperactivation/hyperproliferation, and subsequent lymphadenopathy and autoimmunity. Based on their *in vitro* studies on Fas-deficient *lpr* mice, a hypothesis is proposed for an *in vivo* mechanism for the generation of the *lpr* mouse phenotype, which could potentially lead to therapeutic interventions for related human autoimmune syndromes (Balomenos et al.).

Volpe’s paper, “Fas-Fas Ligand: Checkpoint of T Cell Functions in Multiple Sclerosis,” discusses Fas–Fas ligand interactions, and their involvement in Activation Induced Cell Death (AICD) and negative selection to destroy autoreactive lymphocytes in the context of autoimmune disease and cancer. In particular, this article focuses on the Fas–FasL pathway and how defects in its interaction involving Th17 and Treg can contribute to the pathogenesis of multiple sclerosis (Volpe et al.).

Chakrabandhu’s paper, “Fas Versatile Signaling and Beyond: Pivotal Role of Tyrosine Phosphorylation in Context-Dependent Signaling and Diseases,” discusses the role of the Fas/FasL system as an apoptosis activator as well as a source of non-death signals that can promote survival, proliferation, migrations, and cell invasion. This article focuses on the recent identification of tyrosine phosphorylation in the death domain as a regulator of the reversible signaling switch between the two seemingly opposite roles of Fas/FasL, the factors and context that contribute to the switch, and how pathologies can develop in the signaling pathways (Chakrabandhu and Hueber).

Roberts’s paper, “TNF Blockade Maintains an IL-10+ Phenotype in Human Effector CD4+ and CD8+ Cells,” discusses *in vitro* and *in vivo* data on the regulation of IL-10 expression through TNF blockade and other cytokine/co-stimulatory pathways in CD4+ T cells. The data presented helps provide insight into a previously overlooked mechanism of action for the frequently utilized therapeutic strategy of TNF inhibitors for conditions such as rheumatoid arthritis (Roberts et al.). Interestingly, upregulation of IL-10 as a result of FasL blockade plays a critical role in preventing islet insulitis in NOD mice bearing a loss-of-function gld mutation of FasL (6), which prevents T1D without causing accumulation of the poorly understood Double Negative (DN) T cells (4).

O’Reilly’s paper “The Janus Face of Death Receptor Signaling during Tumor Immunoediting” addresses the issue of tumor cells’ ability to proliferate and evade immune-mediated elimination by resisting the death ligand-induced apoptotic pathway through cleavage and mutations in death ligand receptors, or overexpression of decoy receptors and pro-survival proteins. The article discusses the role of death receptor signaling, and the early elimination and escape phase of tumor immunoediting as it relates to the tumor cells’ ability to resist cell death and metastasize. The article also discusses the opportunities and challenges of developing death receptor agonists for cancer therapeutics (O’Reilly et al.).

In summary, diversity of these studies reveal the complex tasks carried out by TNF superfamily members, which provides a great opportunity for identifying therapeutic targets and at the same time, underlines the challenges.

## Author Contributions

AG and RA read and summarized manuscripts published under the Research topic. AH, HY served as associate editors for the Research Topic and TD read, commented, and edited the editorial.

## Conflict of Interest Statement

The authors declare that the research was conducted in the absence of any commercial or financial relationships that could be construed as a potential conflict of interest.
